# Supplementary material for: Synthesis of a Novel Linear α, ω-Di (Chloro Phosphoramide) Polydimethylsiloxane and Its Applications in Improving Flame-Retardant and Water-Repellent Properties of Cotton Fabrics
Source: Polymers (Basel). 2019 Nov 7;11(11):1829. doi: 10.3390/polym11111829 (PMC6918139; doi:10.3390/polym11111829)
Supplement: Supplementary file 1 [file polymers-11-01829-s001.pdf]

Synthesis of a novel linear α, ω-di (chloro phosphoramide) polydimethylsiloxane and its applications in improving flame-fetardant and water-repellent properties of cotton fabrics

Chaohong Dong, Ling Sun, Xingbo Ma, Zhou Lu *, Pengshuang He, Ping Zhu *

Institute of Functional Textiles and Advanced Materials, State Key Laboratory of Bio-Fibers and Eco-Textiles, College of Textile and Clothing, Qingdao University, Qingdao 266000, China.

Electronic Supplementary information

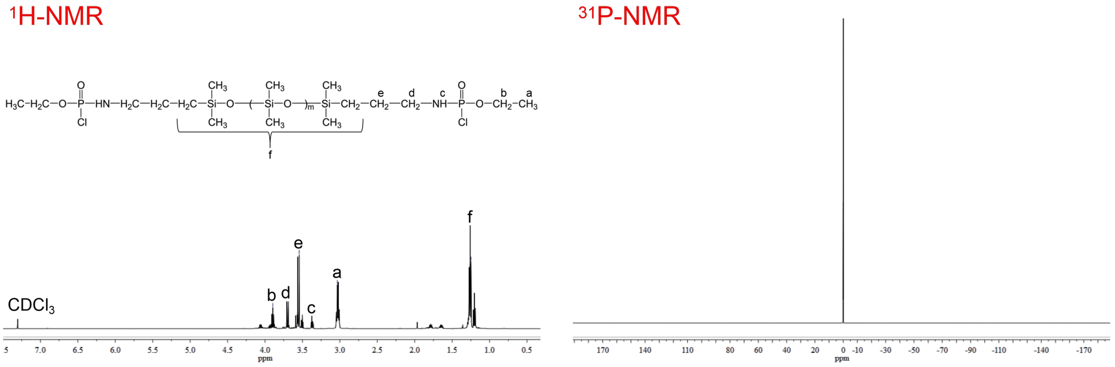

Figure S1. ^{1}H NMR and ^{31}P NMR spectra of CPN-PDMS.

^{1}H NMR and ^{31}P NMR spectrum of CPN-PDMS are presented in Fig. S1. As shown in the ^{1}H NMR spectra, the peaks appeared at 2.95-3.08 ppm, 3.84-3.92 ppm corresponding to $-CH_3$ and $-CH_2-$ of dichloroethyl phosphate. The peaks at 3.65-3.72 ppm, 3.48-3.59 ppm and 1.15-1.36 ppm were attributed to d, e and f (derived from PDMS-2NH_2). Furthermore, the peak of -NH- group connecting dichloroethyl phosphate with PDMS-2NH_2 in the ^{1}H NMR spectra was shown at 3.30-3.41 ppm, which suggests successful reaction happens between dichloroethyl phosphate with PDMS-2NH_2. The single signal at -0.05 ppm in the ^{31}P NMR spectra was corresponded with the unique phosphorus in the CPN-PDMS, indicating that this product has a high purity.
